# Identification of the SARS-CoV-2 Entry Receptor ACE2 as a Direct Target for Transcriptional Repression by Miz1

**DOI:** 10.3389/fimmu.2021.648815

**Published:** 2021-07-07

**Authors:** Jing Yang, Edith A. Perez, Changchun Hou, Pin Zhang, Michelle Van Scoyk, Robert A. Winn, Lijun Rong, Jing Liu

**Affiliations:** ^1^ Department of Surgery, College of Medicine and University of Illinois Cancer Center, University of Illinois at Chicago, Chicago, IL, United States; ^2^ Department of Microbiology and Immunology, College of Medicine, University of Illinois at Chicago, Chicago, IL, United States; ^3^ Massey Cancer Center, Virginia Commonwealth University, Richmond, VA, United States

**Keywords:** COVID-19, SARS-CoV-2 receptor ACE2, transcriptional regulation, COPD, chromatin immunoprecipitation

## Abstract

Multiple lines of evidence have demonstrated that cigarette smoke or Chronic Obstructive Pulmonary Disease upregulates angiotensin-converting enzyme 2, the cellular receptor for the entry of the severe acute respiratory syndrome coronavirus 2, which predisposes individuals to develop severe Coronavirus disease 2019. The reason for this observation is unknown. We recently reported that the loss of function of Miz1 in the lung epithelium in mice leads to a spontaneous COPD-like phenotype, associated with upregulation of angiotensin-converting enzyme 2. We also reported that cigarette smoke exposure downregulates Miz1 in lung epithelial cells and in mice, and Miz1 is also downregulated in the lungs of COPD patients. Here, we provide further evidence that Miz1 directly binds to and represses the promoter of angiotensin-converting enzyme 2 in mouse and human lung epithelial cells. Our data provide a potential molecular mechanism for the upregulation of angiotensin-converting enzyme 2 observed in smokers and COPD patients, with implication in severe Coronavirus disease 2019.

## Introduction

Coronavirus disease 2019 (COVID-19), a newly emerged respiratory disease caused by the severe acute respiratory syndrome coronavirus 2 (SARS-CoV-2), has become pandemic. Most patients with COVID-19 have mild symptoms, but approximately 20% progress to severe diseases, namely, severe pneumonia, acute respiratory distress syndrome, septic shock and/or multiple organ failure driven by hyperinflammation and a cytokine storm syndrome (1, 2). Currently, the mainstay of clinical treatment includes symptomatic management and oxygen therapy, with mechanical ventilation for patients with respiratory failure. Although several drugs are being actively tested, none has been specifically approved for COVID-19 (1, 2).

Human coronaviruses include the two highly pathogenic viruses, SARS-CoV and MERS-CoV, which cause severe respiratory syndrome in humans, and the other four (HCoV-NL63, HCoV-229E, HCoV-OC43 and HKU1), which induce only mild upper respiratory diseases. The newly identified SARS-CoV-2 is closely related to SARS-CoV (3). The genome of SARS-CoV-2 encodes the Spike glycoprotein (S-protein, which contains two subunits, S1 and S2), the envelope protein, the membrane protein, and the nucleocapsid protein. SARS-CoV-S and SARS-CoV-2-S share ∼76% amino acid identity. Entry of coronaviruses into target cells depends on binding of the surface unit, S1, of the S-protein to a cellular receptor, which facilitates viral attachment to the surface of target cells. In addition, entry requires S-protein priming by cellular proteases, which entails S-protein cleavage at the S1/S2 and the S2’ site and allows fusion of viral and cellular membranes, a process driven by the S2 subunit. It has been reported that SARS-CoV-2 uses the SARS-CoV receptor angiotensin-converting enzyme 2 (ACE2), which is abundantly expressed in bronchial and alveolar epithelial cells, for entry and the serine protease TMPRSS2 for S-protein priming (4). Additionally, upon the binding of SARS-CoV to ACE2, desintegrin and metalloproteinase domain 17 (ADAM17) are activated, which in turn mediate proteolysis and ectodomain shedding of ACE2, resulting in the loss of ACE2 at the membrane (5, 6). This has been suggested to contribute to severe acute respiratory failure and comorbidities in COVID-19. Human recombinant soluble ACE2 (hrsACE2) has been demonstrated to block the growth and infection of SARS-CoV-2 and most importantly shows promise for treating severe COVID-19 (7–9). Thus, a better understanding of the regulatory mechanisms that control expression levels of ACE2 could be key to developing effective novel treatments for SARS-CoV-2 infections.

Transcriptional regulation of ACE2 is still poorly understood. The only evidence of transcriptional regulation by the interaction between promoter elements and transcription factors are hepatocyte nuclear factors 1*α*, 1*β*, and 3*β*, which bind to evolutionarily conserved motifs in the proximal ACE2 promoter region in pancreatic islets and kidney cell line (10–12). Androgen and androgen receptor (AR) are reported to upregulate ACE2 transcription, which may explain the gender difference in the susceptibility to COVID-19 in terms of mortality and morbidity (13). Epigenetic induction of ACE2 by cellular stress has also been described in human hepatoma Huh7 cells, which involves the activation of AMP-activated protein kinase and the recruitment of the histone deacetylase SIRT1 (silent information regulator T1) to the conserved upstream regulatory elements of the ACE2 gene (14). Transcriptional repression of ACE2 is also reported to be mediated by the Brahma-related gene-1 (Brg1) chromatin remodeler and forkhead box M1 (FoxM1) transcription factor in cardiac endothelial cells during pathological stress. Transcription factors that regulate ACE2 in the lung have not been identified so far.

We previously reported that Myc interacting zinc finger protein 1 (Miz1; also known as Zbtb17) restrains excessive and persistent pulmonary inflammation through the epigenetic repression of the NF-κB target pro-inflammatory gene CCAAT/Enhancer Binding Protein (*Cebp*) *delta* expression in response to lipopolysaccharide (LPS) or during *Pseudomonas* pneumonia (15). More recently, we reported that mice with lung epithelial cell-specific, but not inflammatory cell-specific, the loss of function of Miz1 develops a spontaneous, age-related progressive COPD-like phenotype, associated with aberrant inflammatory response (16). These mice develop many features that were recapitulated in human COPD. The relevance of our findings to human COPD is supported by our observation that Miz1 protein is downregulated in epithelial cells from explanted lungs of patients with COPD at the time of transplant. In addition, cigarette smoke (CS), the leading cause of COPD, downregulates Miz1 protein in mice and cells (16). Intriguingly, we observed that *Ace2* mRNA levels were remarkably augmented in flow-sorted primary lung epithelial cells isolated from mice with lung epithelial cell-specific loss of function of Miz1 as compared to the control mice (16). Here, we further investigate whether *Ace2* is a direct target for transcriptional repression by Miz1 in both mouse and human lung epithelial cells.

## Materials and Methods

### Reagents

Recombinant mouse TNF was from R&D Systems (410-MT). CS was from Murty Pharmaceuticals.

### Quantitative PCR, ChIP Assay, and ChIP-seq

Quantitative PCR (qPCR) was performed using iQ™ SYBR^®^ Green Supermix (BIO-RAD) on a CFX Connect™ Real-Time PCR Detection System (BIO-RAD). mRNA expression of a particular gene was normalized to hypoxanthine–guanine phosphoribosyltransferse (HPRT) for mouse genes or glyceraldehyde 3-phosphate dehydrogenase (GAPDH) for human genes. Primer sequences were listed in **Supplementary Table 1**. For qRT-PCR, the total RNA was extracted using TRIzol^®^ (Thermo Fisher Scientific), followed by cDNA synthesis using M-MuLV Reverse Transcriptase was according to the manufacturer’s instructions. ChIP assays were done as we previously reported (15). The following antibodies were used for immunoprecipitation: anti-Miz1 (B-10 X, Santa Cruz Biotechnology) and anti-GFP (3E6, Thermo Fisher Scientific). Primer sequences for ChIP-qPCR were listed in **Supplementary Table 2**. For ChIP-seq, DNA samples were quantitated by qubit and sequencing libraries were generated using an Illumina TruSEQ based protocol. Libraries were sequenced on an Illumina NovaSEQ6000 (100 bp, paired-end) at the University of Chicago Genomics Facility and the raw sequencing data were demultiplexed using the Illumina bcl2fastq software. Sequencing raw reads were aligned to the mouse genome mm10 using BWA MEM. Apparent PCR duplicates were removed using Picard MarkDuplicates to ensure that downstream results were not biased by PCR duplication artifacts. Peaks were called against inputs using Macs2; normalized bedgraph tracks were generated using the –SPMR flag, and were converted to bigWig using the UCSC tool bedGraphToBigWig.

### RNA-seq of Primary Lung Epithelial Cells

RNA-seq was performed in flow-sorted primary lung epithelial cells from mice as we reportedly described (16).

### Antibodies for Western Blot

GFP antibody (3E6, Thermo Fisher Scientific), Miz-1 antibody (D7E8B, Cell Signaling Technology) to detect human Miz1 proteins, Miz-1 antibody (PA5-67999, Thermo Fisher Scientific) to detect mouse Miz1 proteins, ACE2 antibody (ab15348, Abcam), and Vinculin antibody (MA5-11690, Invitrogen) were used for Western blot.

### Stable Cell Lines

Miz1 was knocked out in KP cells using the CRISPR-Cas9 system (17). Stable expression of exogenous Miz1 was introduced in Miz1 KO KP cells by lentiviral particle generated by the DNA/RNA Delivery Core of Northwestern University. Miz1 was stably silenced in BEAS-2B, A549, H23, and 2122 cells using the SMARTvector Lentiviral shRNA (Horizon Discovery). Stable expression of exogenous GFP-tagged Miz1 was introduced in BEAS-2B, A549, H23, and 2122 cells by lentiviral particle (ORIGENE; CAT#: RC216143L2V)

### Entry Assay of SARS-Cov-2-Spike Pseudovirus

The entry assay of pseudovirus was performed as described previously (Ref). Briefly, HEK293T cells were co-transfected with an HIV-1 genome plasmid expressing luciferase (pNL4-3.luc.RE) and an expression vector encoding SARS-CoV-2 spike protein using polyethylenimine. Pseudoviruses were harvested 24 h after transfection. Cells were then incubated with pseudoviruses at 37°C for 6 h, and the medium was removed and cells were incubated with fresh culture medium for an additional 48 h. Cells were then washed with PBS and lysed. Luciferase reporter activity was measured using the Neolite Reporter Gene Assay System (PerkinElmer) following manufacturer’s instructions.

### Statistical Analysis

Data were analyzed by an unpaired Student’s *t*-test for comparison between two groups, and one-way ANOVA followed by a Tukey’s *post hoc *test when comparisons of more than two groups are required, or two-way ANOVA followed by a Bonferroni *post hoc* test when multiple comparisons are required.

## Results

### ChIP-seq Reveals Miz1 Binding on the *Ace2* Promoter in Mouse Lung Epithelial Cells

**Figure 1:**
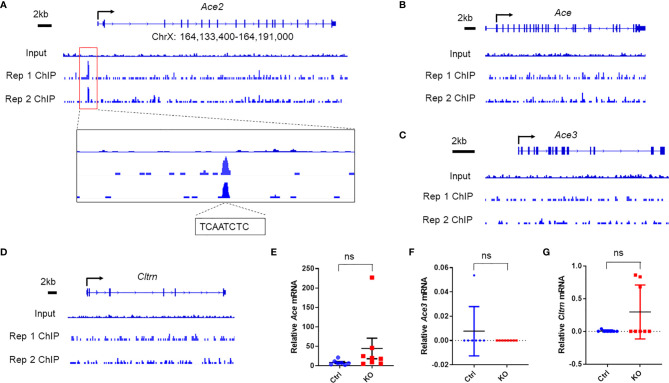
Miz1 specifically binds to the Ace2 promoter in mouse lung epithelial cells as analyzed by ChIP-seq. ChIP-seq traces of Miz1 on the promoters of *Ace2* **(A)**, *Ace* **(B)**, *Ace3* **(C)**, or *Cltrn* **(D)** as indicated. Input is shown as control. Two biological replicates are shown, which represent two independent experiments. In **(A)**, Miz1 binding peak on the *Ace2* promoter is enlarged for viewing and Miz1 binding sequences on the *Ace2* promoter are indicated as “TCAATCTC”. **(E–G)** mRNA expression of *Ace* **(E)**, *Ace3* **(F)**, or *Cltrn* **(G)** by RNA-seq in flow-sorted primary lung epithelial cells isolated from age-matched control *Miz1^fl/fl^* (n = 7) or *SPC-Cre^+^/Miz1^fl/fl^* mice (n = 8). Data are presented as means ± sem. ns, not significant.

We first queried our datasets of high-throughput chromatin immunoprecipitation sequencing (ChIP-seq) with Miz1 antibody for immunoprecipitation (IP) from mouse normal alveolar type II epithelial cell line (MLE-12) under physiological conditions. Several independent reports have demonstrated that Miz1 binds to and represses the promoter of *Cdkn1a* [encoding the cyclin-dependent kinase (CDK) inhibitor p21 (18). Consistent with those reports, our ChIP-seq datasets revealed that Miz1 bound to the *Cdkn1a* promoter [-691 relative to the transcription start site (TSS)], thus validating our ChIP-seq system (**Supplementary Figure 1A**). We and others have reported that the N-terminal poxvirus and zinc-finger domain of Miz1 stabilize its binding to DNA (15). Accordingly, we observed that the binding of the POZ domain deletion mutant of Miz1 [Miz1(δPOZ)] to the *Cdkn1a* promoter was reduced compared to wild-type Miz1 [Miz1(WT)] (**Supplementary Figure 1A**). We also queried our RNA sequencing (RNA-seq) datasets from flow-sorted primary lung epithelial cells isolated from the control mice or mice with lung epithelial cell-specific loss of function of Miz1 (*SPC-Cre^+^/Miz1(POZ)^fl/fl^*) as we reported (16), which was generated by crossing the transgenic mice expressing Cre driven by the human SPC promoter (*SPC-Cre* mice) (19) with *Miz1[7]^fl/fl^* mice, in which *lox*P sites flank the POZ domain of *Miz1* [*Miz1*(δPOZ)] (15). RNA-seq datasets showed that *Cdkn1a* transcription was markedly upregulated in lung epithelial cells isolated from *SPC-Cre^+^/Miz1*(δPOZ)*^fl/fl^* mice compared to the control mice (**Supplementary Figure 1B**), which is in line with our ChIP-seq data (**Supplementary Figure 1A**) and previous reports (18), further validating our ChIP-seq and RNA-seq systems. We then observed that Miz1 bound to the promoter of *Ace2* (-1822 relative to TSS), which contains the Miz1 binding motif YYAN-T/A-YYY (“[ct][ct]A[ctag][at][ct][ct][ct]”) (**Figure 1A**; two biological replicates are shown; Miz1 binding sequence TCAATCTC). The binding of Miz1 to the *Ace2* promoter appears to be specific, as it did not bind to the promoters of the *Ace2* homologs, including angiotensin-converting enzyme (*Ace*), angiotensin-converting enzyme 3 (*Ace3*), or Collectrin (*Cltrn*) (**Figures 1B–D**). Furthermore, we did not observe statistically significant differences in their mRNA expressions between primary lung epithelial cells isolated from the control and *SPC-Cre^+^/Miz1*(δPOZ)*^fl/fl^* mice (**Figures 1E–G**). These unbiased ChIP-seq data suggest that Miz1 specifically binds to the *Ace2* promoter in mouse lung epithelial cells.

### ChIP-Seq Shows That the POZ Domain Is Necessary but Ser178 Phosphorylation Is Dispensable for Miz1 Binding on the *Ace2* Promoter, and TNF Suppresses the Binding

**Figure 2:**
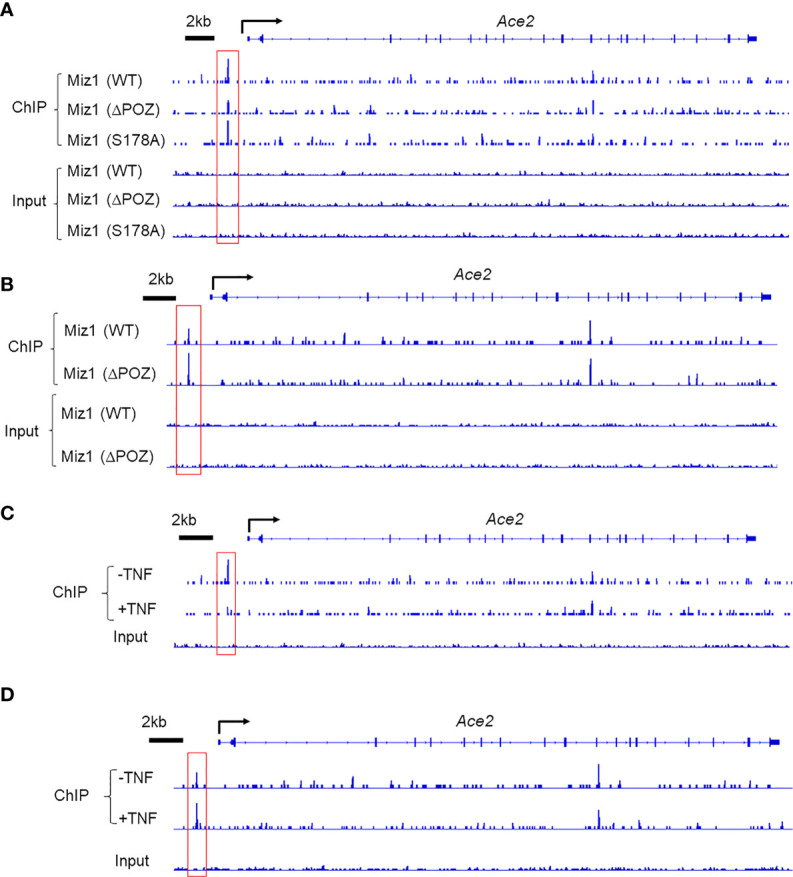
Miz1 binds to the Ace2 promoter in a POZ-domain dependent manner and TNF reduces Miz1 binding in mouse lung epithelial cells as analyzed by ChIP-seq. **(A)** ChIP-seq traces of wild type or mutant Miz1 proteins (δPOZ or S178A) on the *Ace2* promoter. Input is shown as control. **(B)** ChIP-seq traces of RNA Pol II on the *Ace2* promoter in MLE-12/Miz1(WT) and MLE-12/Miz1(δPOZ) cells. Input is shown as control. **(C)** ChIP-seq traces of Miz1 on the *Ace2* promoter in TNF-non-treated or -treated MLE-12/Miz1(WT) cells. Input is shown as control. **(D)** ChIP-seq traces of RNA Pol II on the *Ace2* promoter in TNF-non-treated or -treated MLE-12/Miz1(WT)cells. Input is shown as control.

Consistent with the role of the POZ domain in Miz1 binding to DNA, the binding of Miz1(δPOZ) to the *Ace2* promoter was decreased compared to Miz1(WT), as revealed by Miz1 ChIP-seq in stable MLE-12 cell lines, in which endogenous Miz1 was stably knocked down by shRNA while exogenous wild-type or POZ domain deletion mutant of Miz1 (WT or δPOZ) was stably expressed as we reported previously (16) (**Figure 2A**). RNA polymerase II (RNA Pol II) ChIP-seq revealed an increased RNA Pol II recruitment on the *Ace2* promoter in MLE-12/Miz1(δPOZ) cells compared to MLE-12/Miz1(WT) cells (**Figure 2B**), indicating an increased transcription by loss of the Miz1 POZ domain. These data are in line with our previous report that *Ace2* was upregulated in flow-sorted primary lung epithelial cells isolated from mice with lung epithelial cell-specific loss of the Miz1 POZ domain as compared to the control mice (16). We previously reported that Ser178 phosphorylation of Miz1 is required for Miz1 to repress the *Cebpd* promoter (15). We noticed that mutation of Ser178 to the non-phosphorylatable alanine did not interfere with Miz1 binding on the *Ace2* promoter (**Figure 2A**), suggesting Ser178 is dispensable for Miz1 binding to the *Ace2* promoter. Interestingly, we observed that in response to TNF, the binding of Miz1 to the *Ace2* promoter was reduced (**Figure 2C**), accompanied with enhanced recruitment of RNA Pol II (**Figure 2D**). These data imply that the repression of the *Ace2* promoter by Miz1 might be relieved upon TNF simulation, which are in line with previous reports that *Ace2* expression is elevated by TNF or other inflammatory stimuli (20).

### ChIP-qPCR Confirms Miz1 Binding on the *Ace2* Promoter in Mouse and Human Lung Epithelial Cells

**Figure 3:**
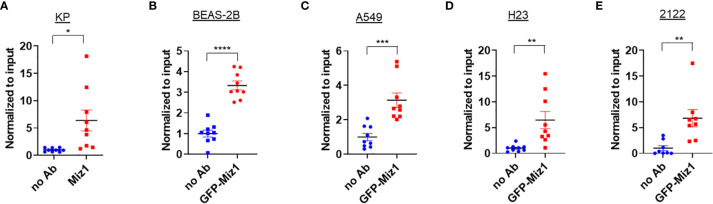
Miz1 binds to the Ace2 promoter in both mouse and human lung epithelial cells as analyzed by ChIP-qPCR. **(A–E)** ChIP-qPCR shows enrichment of Miz1 binding on the *Ace2* promoter as compared to no antibody (no Ab) control in KP **(A)**, BEAS-2B **(B)**, A549 **(C)**, H23 **(D)**, and 2122 **(E)** cells. Data are presented as means ± sem from three independent experiments. *p < 0.05, **p < 0.01, ***p < 0.001 and ****p < 0.0001.

We validated our Miz1 ChIP-seq data by ChIP coupled with quantitative PCR (qPCR). An enrichment of endogenous Miz1 binding on the *Ace2* promoter was apparent in mouse lung epithelial cells (**Figure 3A**). To determine whether Miz1 also binds to the *ACE2* promoter in human lung epithelial cells, we used stable normal bronchial epithelial BEAS-2B cells, adenocarcinomic human alveolar basal epithelial A549 cells, and adenocarcinomic human lung epithelial H23 and 2122 cells available in our laboratory, in which exogenous green fluorescent protein (GFP)-tagged Miz1 was stably expressed. ChIP-qPCR showed GFP-Miz1 binds at the *ACE2* promoter in BEAS-2B (**Figure 3B**), A549 (**Figure 3C**), H23 (**Figure 3D**), and 2122 cells (**Figure 3E**). These data further confirmed that Miz1 binds to the *ACE2* promoter in both mouse and human lung epithelial cells.

### Knockout of Miz1 Augments Ace2 Expression in Mouse Lung Epithelial Cells, Which Is Reversed by Complementation of Exogenous Miz1

**Figure 4:**
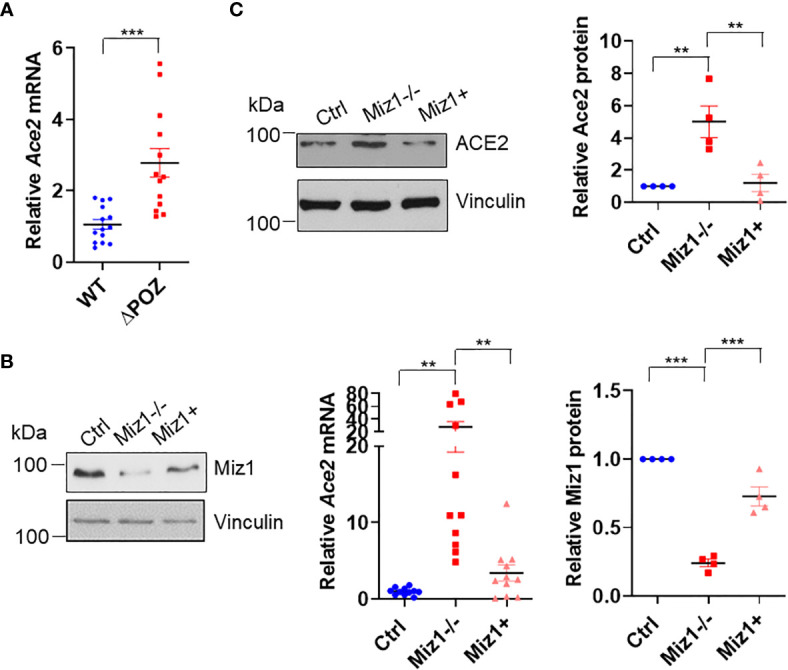
Miz1 represses Ace2 transcription in mouse lung epithelial cells. **(A)** *Ace2* mRNA expression in MLE-12/Miz1(WT) and MLE-12/Miz1(δPOZ) cells as analyzed by RT-qPCR. **(B, C)** *Ace2* mRNA **(B)** and protein expression **(C)** in the control KP cells (Ctrl), Miz1 KO KP cells (Miz1^−/−^) or Miz1 KO KP cells expressing exogenous Miz1 (Miz1+). Data are presented as means ± sem from three to four independent experiments. *p < 0.05, **p < 0.01, ***p < 0.001 and ****p < 0.0001.

Consistent with the data that there was an increased RNA Pol II recruitment on the *Ace2* promoter in MLE-12/Miz1(δPOZ) cells compared to MLE-12/Miz1(WT) cells (**Figure 2B**), *Ace2* mRNA levels were augmented in MLE-12/Miz1(δPOZ) cells compared to MLE-12/Miz1(WT) cells (**Figure 4A**). Moreover, we took advantage of the stable Miz1 knockout (KO) cells available in our laboratory, which was established using Crispr/Cas 9 system in mouse lung adenocarcinoma cells originated from tumors of Cre recombinase-treated Kras^LSL-G12D/+^Trp53^fl/f^ mice, which carries a lox-stop-lox (LSL) sequence followed by the KRAS G12D point mutation allele as well as loxP sites flanking exons 2–10 of the transformation related protein 53 (*Trp53*) gene as reported (referred to hereafter as KP cells) (21, 22). These cells have been reported to be of alveolar epithelial type II cells origin (23). Knockout of Miz1 drastically increased *Ace2* mRNA and protein expressions in KP cells, which was rescued by re-introduction of exogenous Miz1 (**Figures 4B, C**). Together, our data suggest that *Ace2* is a direct target for transcriptional repression by Miz1 in mouse lung epithelial cells.

### Knockdown of MIZ1 Also Increases While Overexpression of MIZ1 Decreases ACE2 Expression in Human Lung Epithelial Cells

**Figure 5:**
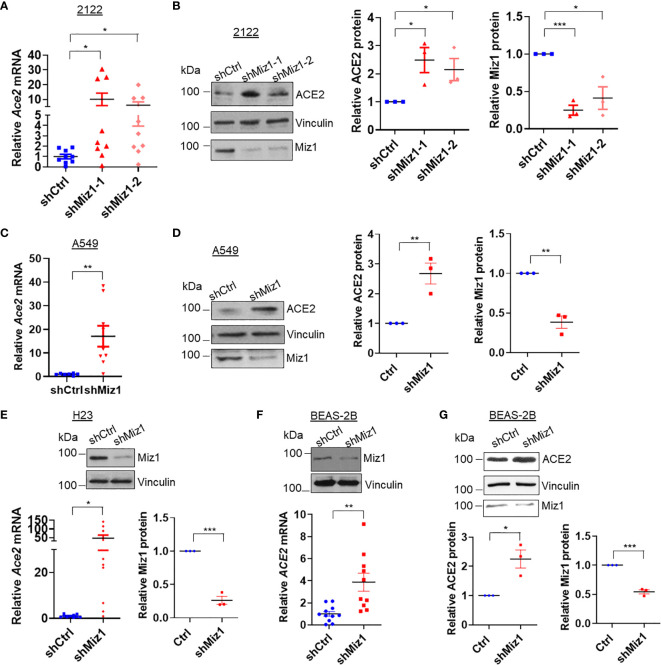
Knockdown of MIZ1 also increases ACE2 expression in human lung epithelial cells. **(A, C, E, F)** *ACE2* mRNA levels in the parental 2122, A549, H23, or BEAS-2B cells, or their corresponding cells expressing control shRNA (shCtrl) or shRNA(s) against Miz1 (shMiz1). **(B, D, G)** Protein expression of ACE2, Vinculin, and Miz1 in 2122, A549, or BEAS-2B cells stably expressing shCtrl or shRNA(s) against Miz1. Data are presented as means ± sem from three independent experiments. *p < 0.05, **p < 0.01 and ***p < 0.001.

**Figure 6:**
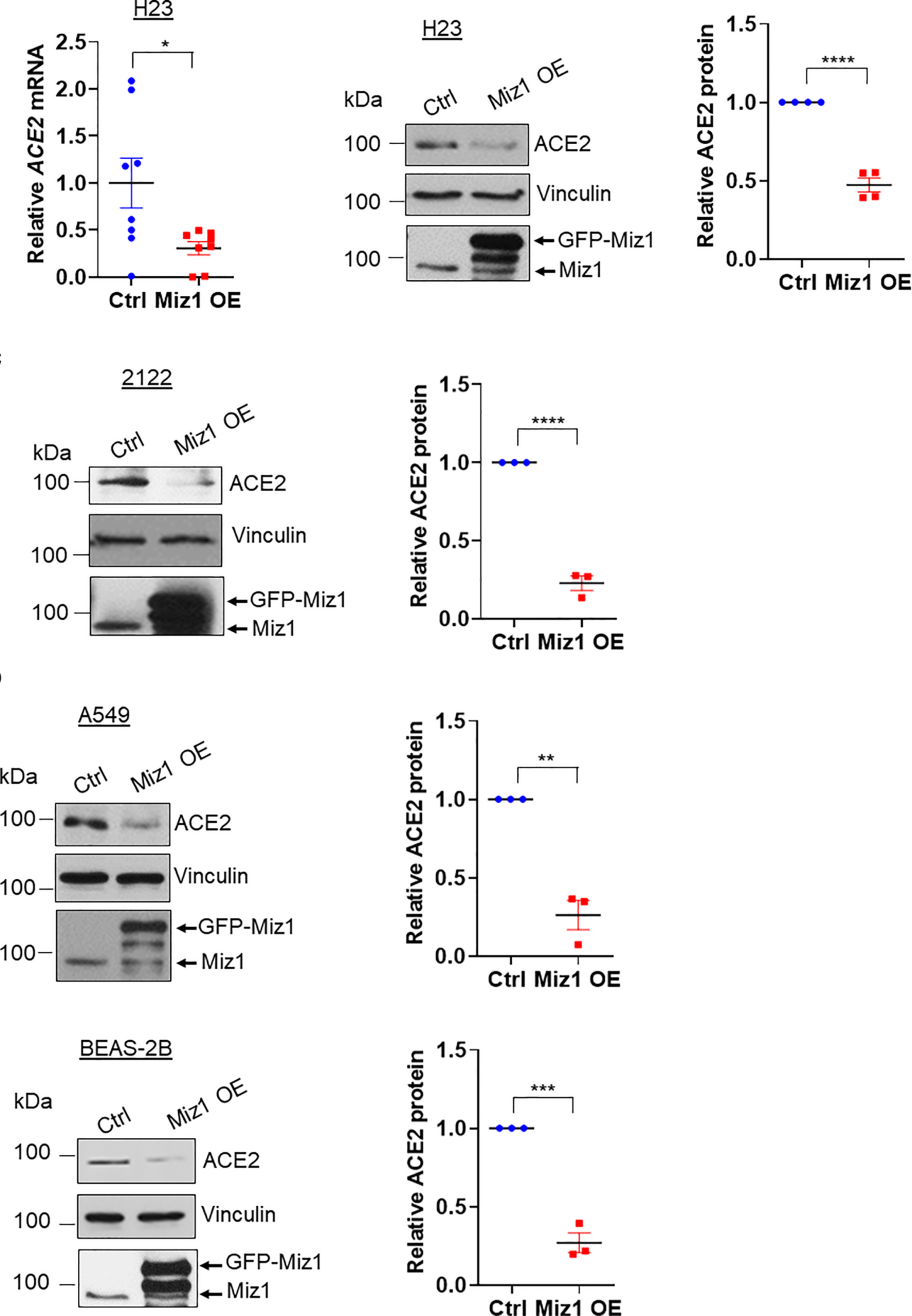
Knockdown of MIZ1 reduces ACE2 expression in human lung epithelial cells. **(A)** *ACE2* mRNA in H23 cells stably expressing GFP-tagged human MIZ1 protein. **(B–E)** ACE2 protein expression in H23 **(B)**, 2122 **(C)**, A549 **(D)**, and BEAS-2B cells **(E)** stably expressing GFP-tagged human MIZ1 protein. Data are presented as means ± sem from three to four independent experiments. *p < 0.05, **p < 0.01, ***p < 0.001 and ****p < 0.0001.

Next we sought to determine whether a Miz1-mediated repression of *Ace2* transcription also occurs in human lung epithelial cells. We generated stable cell lines expressing shRNA against Miz1 or overexpressing exogenous GFP-tagged human Miz1 protein in human lung epithelial cell lines BEAS-2B, A549, H23 and 2122, respectively. The silencing of Miz1 with two shRNAs targeting different regions of the Miz1 gene in 2122 cells upregulated *Ace2* mRNA and protein expressions (**Figures 5A, B**). Similar results were obtained in A549 (**Figures 5C, D**), H23 (**Figure 5E**), and BEAS-2B cells (**Figures 5F, G**). Conversely, overexpression of exogenous Miz1 decreased ACE2 expression in H23 (**Figures 6A, B**), 2122 (**Figure 6C**), A549 cells (**Figure 6D**), and BEAS-2B cells (**Figure 6E**). These data suggest that the repression of *Ace2* by Miz1 is a general mechanism in both human and mouse lung epithelial cells.

### Loss of Function of Miz1 Augments Ace2 Expression in Response to TNF or Cigarette Smoke

**Figure 7:**
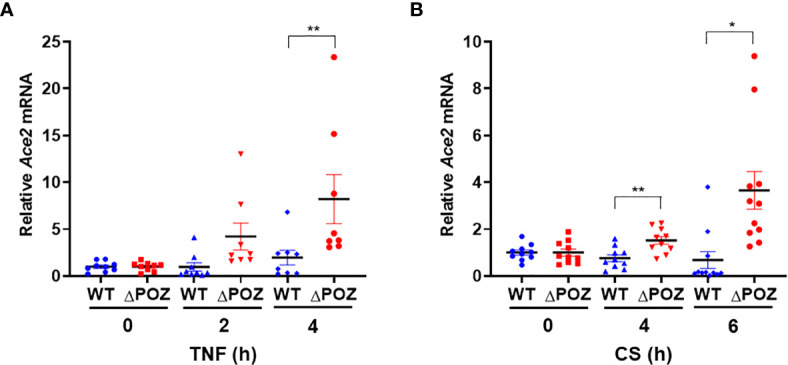
Loss of function of MIZ1 augments Ace2 expression in response to TNF or CS. **(A, B)** *Ace2* mRNA levels in TNF- **(A)** or CS-treated **(B)** MLE-12/Miz1(WT) and MLE-12/Miz1(δPOZ) cells as indicated. Data are presented as means ± sem (fold changes normalized to corresponding non-treated controls) from three independent experiments. TNF, 5 ng/ml. CS, 100 µg/ml. *p < 0.05 and **p < 0.01.

Our data showed that the loss of function of Miz1 enhances Ace2 expression under resting conditions in lung epithelial cells. We investigated whether Miz1 also affects Ace2 expression in response to inflammatory stimuli, such as TNF, or cigarette smoke, the most environmental risk factor for COPD. MLE-12/Miz1(WT) or MLE-12/Miz1(δPOZ) cells were treated with TNF for 2 or 4 h. While *Ace2* was modestly upregulated by TNF in MLE-12/Miz1(WT) cells, its mRNA expression was higher in MLE-12/Miz1(δPOZ) cells compared to MLE-12/Miz1(WT) in response to TNF (**Figure 7A**). Similar results were obtained with CS treatment (**Figure 7B**). These data suggest that Miz1 represses Ace2 under basal conditions as well as in response to TNF or CS.

As our data showed that the loss of function or knockout/knockdown of Miz1 augments the expression of Ace2, the host cell receptor for SARS-CoV-2, we sought to determine the effect of Miz1 on SARS-CoV-2 spike protein-mediated entry using HIV-based pseudovirus assay. Stable H23 cells expressing control shRNA or Miz1 shRNA were incubated with SARS-CoV-2 spike protein pseudoviruses (24), and luciferase reporter assay showed that the entry efficiency of SARS-CoV-2 spike protein pseudoviruses was enhanced by silencing of Miz1 (**Supplementary Figure 2**). These results provide evidence to support our conclusion that Ace2 is upregulated by loss of function or knockout/knockdown of Miz1.

## Discussion

While whether COVID-19 has a greater incidence in smokers than non-smokers is contradictory and inconclusive thus far, multiple lines of evidence have demonstrated that cigarette smoke (CS) or Chronic Obstructive Pulmonary Disease (COPD) predisposes individuals to develop severe symptoms of COVID-19 disease (25–30). A systemic review and meta-analysis of a total of 2,002 COVID-19 cases revealed that the presence of COPD is associated with a nearly 4-fold higher risk of developing severe COVID-19 (25, 26). Independent studies also showed that COPD is a strong predictor of poor outcome in elderly patients (27). The reason for this observation is unknown. An understanding of the underlying mechanisms is urgently needed to develop therapies for disease prevention and treatment.

Accumulating evidence reveals that CS exposure upregulates ACE2, and ACE2 has been found to be significantly upregulated in smokers compared with non-smokers, as well as in patients with COPD compared with healthy subjects (20, 31–36). This has been suggested to contribute to severe COVID-19 observed in smokers and COPD patients (20, 31, 32). However, the underlying mechanism by which ACE2 is upregulated in smokers and COPD patients is not known. We recently reported that CS exposure downregulates Miz1 in lung epithelial cells and in mice, and Miz1 is also downregulated in the lungs from COPD patients (15). Importantly, loss of function of Miz1 in the lung epithelium in mice leads to a spontaneous COPD-like phenotype, associated with upregulation of ACE2 (15). Here, we provide further evidence that Miz1 directly binds to and represses the promoter of *Ace2* in a POZ domain-dependent manner. Collectively, our data provide a potential molecular mechanism for the upregulation of ACE2 in smokers and COPD patients through downregulation of Miz1, with implication in severe COVID-19.

While most of the studies to date have demonstrated an association between smoking and more severe symptoms of COVID-19, there are reports that have suggested there is no or even an inverse relationship between smoking and COVID-19 prevalence (29, 37). The controversies were likely due to the challenges in the studies on COVID-19, including insufficient sample sizes with a wider range of geographical settings, heterogeneity in respect of smoking status, and inadequate statistical control for confounding factors such as age, gender and co-morbidities. Nonetheless, while data on the association between smoking and COVID-19 prevalence is mixed, the available evidence suggests that smoking is associated with an increased severity of disease and mortality in hospitalized COVID-19 patients. As we are still in the early stages of understanding the pathogenesis of COVID-19, more extensive research is required to validate the initial findings and also to establish whether and how an increased ACE2 expression contributes to the initiation and progression of COVID-19 in smokers and COPD patients.

The current COVID-19 pandemic has put a great pressure on the healthcare system around the world. Identification of the risk factors and the underlying mechanisms is crucial especially for the severe form of the disease. Hyperinflammation and cytokine storm have been considered as a major cause of mortality in COVID-19 (1, 2). We have reported that the loss of function of Miz1 leads to an exacerbated inflammatory cytokine production under basal conditions and in response to inflammatory stimuli (15). In addition, we recently reported that the downregulation of Miz1 by CS exposure or in COPD patients is attributed to the upregulation of the Miz1 E3 ubiquitin ligase Mule [Mcl-1 (myeloid cell leukemia 1) ubiquitin ligase E3; also known as Huwe1] (38). Therefore, targeting the Mule-Miz1 axis might provide potential target in the prevention and treatment of COVID-19 by dual mechanisms: downregulating ACE2 and suppressing the inflammatory response.

## Data Availability Statement

The datasets presented in this study can be found in online repositories. The names of the repository/repositories and accession number(s) can be found below: https://github.com/jinglius815/RNA-seq.

## Author Contributions

JY, CH, EP, PZ, and MS performed experiments and analyzed data. JL supervised the study and wrote the manuscript. LR provided reagents and suggestions, and edited the manuscript. RW provided suggestions and reagents. All authors contributed to the article and approved the submitted version.

## Funding

JL is supported by the US National Institutes of Health (HL141459 to JL) and Chicago Biomedical Consortium (CBC) COVID-19 Response Award.

## Conflict of Interest

The authors declare that the research was conducted in the absence of any commercial or financial relationships that could be construed as a potential conflict of interest.

## Supplementary Material

Supplementary Figure 1
**(A)** ChIP-seq traces of Miz1 (WT or δPOZ) proteins on the *Cdkn1a* promoter. Input is shown as control. **(B)** mRNA expression of *Cdkn1a* by RNA-seq in flow-sorted primary lung epithelial cells isolated from age-matched control *Miz1[7]^fl/fl^* (n = 3) or *SPC-Cre^+^/Miz1[7]^fl/fl^* mice (n = 4). Data are presented as means ± sem. ***p* < 0.01.

Supplementary Figure 2 Entry efficiency of SARS-CoV-2-spike pseudoviruses into H23 cells stably expressing shCtrl or shMiz1. n = 5-6 biological replicates. Entry efficiency was determined by background (no pseudoviruses)-subtracted luciferase activity. **p* < 0.05.

## References

[1] Mehta P McAuley DF Brown M Sanchez E Tattersall RS Manson JJ. COVID-19: Consider Cytokine Storm Syndromes and Immunosuppression. Lancet (2020) 395(10229):1033–4. 10.1016/S0140-6736(20)30628-0 PMC7270045 32192578

[2] Stebbing J Phelan A Griffin I Tucker C Oechsle O Smith D. COVID-19: Combining Antiviral and Anti-Inflammatory Treatments. Lancet Infect Dis (2020) 20(4):400–2. 10.1016/S1473-3099(20)30132-8 PMC7158903 32113509

[3] Zhu N Zhang D Wang W Li X Yang B Song J. A Novel Coronavirus From Patients With Pneumonia in China, 2019. N. Engl J Med (2020) 382(8):727–33. 10.1056/NEJMoa2001017 PMC7092803 31978945

[4] Hoffmann M Kleine-Weber, H Schroeder S Kruger N Herrler T Erichsen S. SARS-Cov-2 Cell Entry Depends on ACE2 and TMPRSS2 and is Blocked by a Clinically Proven Protease Inhibitor. Cell (2020) 181(2):271–80.e8. 10.1016/j.cell.2020.02.052 32142651 PMC7102627

[5] Lambert DW Yarski M Warner FJ Thornhill P Parkin ET Smith AI. Tumor Necrosis Factor-Alpha Convertase (ADAM17) Mediates Regulated Ectodomain Shedding of the Severe-Acute Respiratory Syndrome-Coronavirus (SARS-Cov) Receptor, Angiotensin-Converting Enzyme-2 (ACE2). J Biol Chem (2005) 280(34):30113–9. 10.1074/jbc.M505111200 PMC8062222 15983030

[6] Kuba K Imai Y Rao S Gao H Guo F Guan B. A Crucial Role of Angiotensin Converting Enzyme 2 (ACE2) in SARS Coronavirus-Induced Lung Injury. Nat Med (2005) 11(8):875–9. 10.1038/nm1267 PMC7095783 16007097

[7] Monteil V Kwon H Prado P Hagelkrüys A Wimmer RA Stahl M. Inhibition of SARS-Cov-2 Infections in Engineered Human Tissues Using Clinical-Grade Soluble Human ACE2. Cell (2020) 181(4):905–13.e7. 10.1016/j.cell.2020.04.004 32333836 PMC7181998

[8] Abd El-Aziz TM Al-Sabi A Stockand JD. Human Recombinant Soluble ACE2 (Hrsace2) Shows Promise for Treating Severe COVID­19. Signal Transduct Target Ther (2020) 5(1):258. 10.1038/s41392-020-00374-6 33144565 PMC7607365

[9] Zoufaly A Poglitsch M Aberle JH Hoepler W Seitz T Traugott M. Human Recombinant Soluble ACE2 in Severe COVID-19. Lancet Respir Med (2020) 8(11):1154–8. 10.1016/S2213-2600(20)30418-5 PMC7515587 33131609

[10] Senkel S Lucas B Klein-Hitpass L Ryffel GU. Identification of Target Genes of the Transcription Factor HNF1beta and HNF1alpha in a Human Embryonic Kidney Cell Line. Biochim Biophys Acta (2005) 1731(3):179–90. 10.1016/j.bbaexp.2005.10.003 16297991

[11] Pedersen KB Chodavarapu H Lazartigues E. Forkhead Box Transcription Factors of the FOXA Class are Required for Basal Transcription of Angiotensin-Converting Enzyme 2. J Endocr Soc (2017) 1(4):370–84. 10.1210/js.2016-1071 PMC5656262 29082356

[12] Pedersen KB Chhabra KH Nguyen VK Xia H Lazartigues E. The Transcription Factor HNF1α Induces Expression of Angiotensin-Converting Enzyme 2 (ACE2) in Pancreatic Islets From Evolutionarily Conserved Promoter Motifs. Biochim Biophys Acta (2013) 1829(11):1225–35. 10.1016/j.bbagrm.2013.09.007 PMC3838857 24100303

[13] Qiao Y Wang X-M Mannan R Pitchiaya S Zhang Y Wotring JW. Targeting Transcriptional Regulation of SARS-Cov-2 Entry Factors ACE2 and TMPRSS2. Proc Natl Acad Sci (2021) 118(1):e2021450118. 10.1073/pnas.2021450118 PMC7817128 33310900

[14] Clarke NE Belyaev ND Lambert DW Turner AJ. Epigenetic Regulation of Angiotensin-Converting Enzyme 2 (ACE2) by SIRT1 Under Conditions of Cell Energy Stress. Clin Sci (Lond) (2014) 126(7):507–16. 10.1042/CS20130291 24147777

[15] Do-Umehara HC Chen C Urich D Zhou L Qiu J Jang S. Suppression of Inflammation and Acute Lung Injury by Miz1 via Repression of C/EBP-Delta. Nat Immunol (2013) 14(5):461–9. 10.1038/ni.2566 PMC3631447 23525087

[16] Do-Umehara HC Chen C Zhang Q Misharin AV Abdala–Valencia H Casalino–Matsuda SM. Epithelial Cell-Specific Loss of Function of Miz1 Causes a Spontaneous COPD-Like Phenotype and Up-Regulates Ace2 Expression in Mice. Sci Adv (2020) 6(33):eabb7238. 10.1126/sciadv.abb7238 32851183 PMC7428331

[17] Ran FA Hsu PD Wright J Agarwala V Scott DA Zhang F. Genome Engineering Using the CRISPR-Cas9 System. Nat Protoc (2013) 8(11):2281–308. 10.1038/nprot.2013.143 PMC3969860 24157548

[18] Seoane J Le H-V Massagué J. Myc Suppression of the p21Cip1 Cdk Inhibitor Influences the Outcome of the P53 Response to DNA Damage. Nature (2002) 419(6908):729–34. 10.1038/nature01119 12384701

[19] Okubo T Knoepfler PS Eisenman RN Hogan BL. Nmyc Plays an Essential Role During Lung Development as a Dosage-Sensitive Regulator of Progenitor Cell Proliferation and Differentiation. Development (2005) 132(6):1363–74. 10.1242/dev.01678 15716345

[20] Smith JC Sausville EL Girish V Yuan ML Vasudevan A John KM. Cigarette Smoke Exposure and Inflammatory Signaling Increase the Expression of the SARS-Cov-2 Receptor ACE2 in the Respiratory Tract. Dev Cell (2020) 53(5):514–529.e3. 10.1016/j.devcel.2020.05.012 32425701 PMC7229915

[21] Glasauer A Sena LA Diebold LP Mazar AP Chandel NS. Targeting SOD1 Reduces Experimental non–Small-Cell Lung Cancer. J Clin Invest (2014) 124(1):117–28. 10.1172/JCI71714 PMC3871252 24292713

[22] DuPage M Dooley AL Jacks T. Conditional Mouse Lung Cancer Models Using Adenoviral or Lentiviral Delivery of Cre Recombinase. Nat Protoc (2009) 4(7):1064–72. 10.1038/nprot.2009.95 PMC2757265 19561589

[23] Xu X Rock JR Lu Y Futtner C Schwab B Guinney J. Evidence for Type II Cells as Cells of Origin of K-Ras-Induced Distal Lung Adenocarcinoma. Proc Natl Acad Sci USA (2012) 109(13):4910–5. 10.1073/pnas.1112499109 PMC3323959 22411819

[24] Du R Cooper L Chen Z Lee H Rong L Cui Q. Discovery of Chebulagic Acid and Punicalagin as Novel Allosteric Inhibitors of SARS-Cov-2 3clpro. Antiviral Res (2021) p:105075. 10.1016/j.antiviral.2021.105075 PMC8052511 33872675

[25] Alqahtani JS Oyelade T Aldhahir AM Alghamdi SM Almehmadi M Alqahtani AS. Prevalence, Severity and Mortality Associated With COPD and Smoking in Patients With COVID-19: A Rapid Systematic Review and Meta-Analysis. PloS One (2020) 15(5):e0233147. 10.1371/journal.pone.0233147 32392262 PMC7213702

[26] Zhao Q Meng M Kumar R Wu Y Huang J Lian N. The Impact of COPD and Smoking History on the Severity of Covid-19: A Systemic Review and Meta-Analysis. J Med Virol (2020). 10.1002/jmv.25889 PMC7262275 32293753

[27] Wang L He X Yu D Hu M Bao H Liu J. Coronavirus Disease 2019 in Elderly Patients: Characteristics and Prognostic Factors Based on 4-Week Follow-Up. J Infect (2020). 10.1016/j.jinf.2020.03.019 PMC7118526 32240670

[28] Peng F Lei S Zhang Q Zhong Y Wu S. Smoking is Correlated With the Prognosis of Coronavirus Disease 2019 (COVID-19) Patients: An Observational Study. Front Physiol (2021) 12:634842. 10.3389/fphys.2021.634842 33762967 PMC7982916

[29] Shastri MD Shukla SD Chong WC Kc R Dua K Patel RP. Smoking and COVID-19: What We Know So Far. Respir Med (2021) 176:106237. 10.1016/j.rmed.2020.106237 33246296 PMC7674982

[30] Guan WJ Ni ZY Hu Y Liang WH Ou CQ He JX. Clinical Characteristics of Coronavirus Disease 2019 in China. N Engl J Med (2020) 382(18):1708–20. 10.1056/NEJMoa2002032 PMC7092819 32109013

[31] Leung JM Yang CX Tam A Shaipanich T Hackett TL Singhera GK. ACE-2 Expression in the Small Airway Epithelia of Smokers and COPD Patients: Implications for COVID-19. Eur Respir J (2020) 55(5). 10.1183/13993003.00688-2020 PMC7144263 32269089

[32] Cai G Bossé Y Xiao F Kheradmand F Amos CI. Tobacco Smoking Increases the Lung Gene Expression of ACE2, the Receptor of SARS-Cov-2. Am J Respir Crit Care Med (2020) 201(12):1557–9. 10.1164/rccm.202003-0693LE PMC7301735 32329629

[33] Russo P Bonassi S Giacconi R Malavolta M Tomino C Maggi F. COVID-19 and Smoking: Is Nicotine the Hidden Link? Eur Respir J (2020) 55(6). 10.1183/13993003.01116-2020 PMC7236819 32341101

[34] Zhang H Rostami MR Leopold PL Mezey JG O'Beirne SL Strulovici–Barel Y. Expression of the SARS-Cov-2 ACE2 Receptor in the Human Airway Epithelium. Am J Respir Crit Care Med (2020) 202(2):219–29. 10.1164/rccm.202003-0541OC PMC7365377 32432483

[35] Leung JM Yang CX Sin DD. COVID-19 and Nicotine as a Mediator of ACE-2. Eur Respir J (2020) 55(6). 10.1183/13993003.01261-2020 PMC7191112 32350104

[36] Olds JL Kabbani N. Is Nicotine Exposure Linked to Cardiopulmonary Vulnerability to COVID-19 in the General Population? FEBS J (2020) 287(17):3651–5. 10.1111/febs.15303 PMC7228237 32189428

[37] Leung JM Sin DD. Smoking, ACE-2 and COVID-19: Ongoing Controversies. Eur Respir J (2020) 56(1). 10.1183/13993003.01759-2020 PMC7363948 32430431

[38] Yang Y Do H Tian X Zhang C Liu X Dada LA. E3 Ubiquitin Ligase Mule Ubiquitinates Miz1 and is Required for Tnfalpha-Induced JNK Activation. Proc Natl Acad Sci USA (2010) 107(30):13444–9. 10.1073/pnas.0913690107 PMC2922175 20624960

