# Supplementary figures and images for: Identification of the SARS-CoV-2 Entry Receptor ACE2 as a Direct Target for Transcriptional Repression by Miz1
Source: Front Immunol. 2021 Jul 7;12:648815. doi: 10.3389/fimmu.2021.648815 (PMC8292894; doi:10.3389/fimmu.2021.648815)

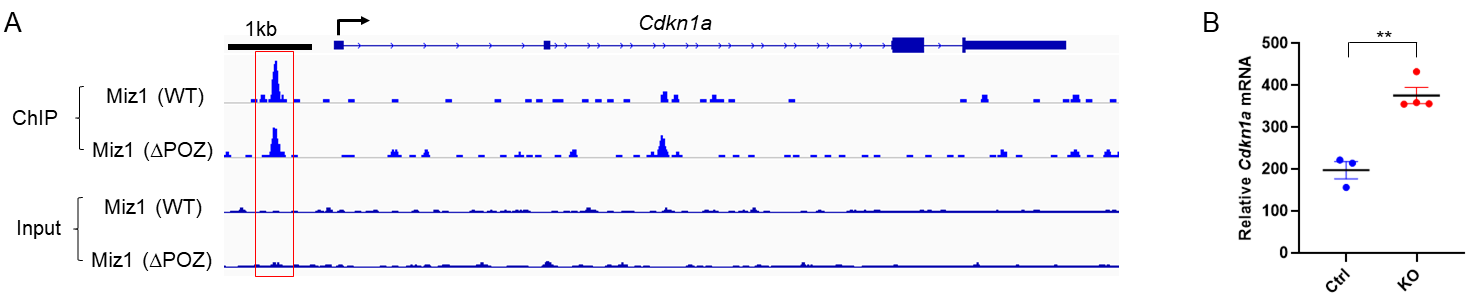

Supplement: Supplementary Figure 1 — (A) ChIP-seq traces of Miz1 (WT or δPOZ) proteins on the Cdkn1a promoter. Input is shown as control. (B) mRNA expression of Cdkn1a by RNA-seq in flow-sorted primary lung epithelial cells isolated from age-matched control Miz1[7]fl/fl (n = 3) or SPC-Cre+/Miz1[7]fl/fl mice (n = 4). Data are presented as means ± sem. **p < 0.01. [file Image_1.tif]

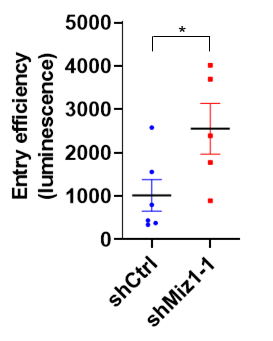

Supplement: Supplementary Figure 2 — Entry efficiency of SARS-CoV-2-spike pseudoviruses into H23 cells stably expressing shCtrl or shMiz1. n = 5-6 biological replicates. Entry efficiency was determined by background (no pseudoviruses)-subtracted luciferase activity. *p < 0.05. [file Image_2.tif]
