# Supplementary material for: Identification of the SARS-CoV-2 Entry Receptor ACE2 as a Direct Target for Transcriptional Repression by Miz1
Source: Front Immunol. 2021 Jul 7;12:648815. doi: 10.3389/fimmu.2021.648815 (PMC8292894; doi:10.3389/fimmu.2021.648815)
Supplement: Supplementary file 3 [file Table_1.docx]

**Supplementary Table 1**

Mouse HPRT:

5'-AGGCCAGACTTTGTTGGATTTGAA-3'

5'-CAACTTGCGCTCATCTTAGGCTTT-3'

Human GAPDH:

5’- GAA GGT GAA GGT CGG AGT C -3’

5’- GAA GAT GGT GAT GGG ATT TC  -3’

Mouse Ace2:

5’- GCCATCTCGTTTTTCAGGACC-3’

5’- GGCGACAAGCACAGACTACAA-3’

Human Ace2:

5’- TGAGAGCACTGAAGACCCATT-3’

5’- ACAGTCCACACTTGCCCAAAT-3’

**Supplementary Table 2**

Mouse Ace2:

5’- CACCCTCCTCCTCCAGTGTA-3’

5’- AAACAAGCTGCTGGACCAAT-3’

Human Ace2:

5’- AACCCAAGTTCAAAGGCTGA-3’

5’- CCAAAAATGTCTTGGCCTGT-3’
